# Transcriptomic Profiling Reveals That HMGB1 Induces Macrophage Polarization Different from Classical M1

**DOI:** 10.3390/biom12060779

**Published:** 2022-06-02

**Authors:** Heshuang Qu, Rebecka Heinbäck, Henna Salo, Ewoud Ewing, Alexander Espinosa, Cecilia Aulin, Helena Erlandsson Harris

**Affiliations:** 1 Department of Medicine, Solna, Karolinska Institutet, and Centre for Molecular Medicine, Karolinska University Hospital, 171 64 Stockholm, Sweden; heshuang.qu@ki.se (H.Q.); rebecka.heinback@ki.se (R.H.); henna.e.salo@gmail.com (H.S.); alexander.espinosa@ki.se (A.E.); cecilia.aulin@ki.se (C.A.); 2 Department of Clinical Neuroscience, Karolinska Institutet, and Centre for Molecular Medicine, Karolinska University Hospital, 171 64 Stockholm, Sweden; ewoud.ewing@ki.se; 3 Broegelmann Research Laboratory, Department of Clinical Science, University of Bergen, 5021 Bergen, Norway

**Keywords:** HMGB1, RNA sequencing, macrophage polarization, gene ontology, foam cell formation

## Abstract

Macrophages are key inflammatory immune cells that display dynamic phenotypes and functions in response to their local microenvironment. In different conditions, macrophage polarization can be induced by high-mobility group box 1 (HMGB1), a nuclear DNA-binding protein that activates innate immunity via the Toll-like receptor (TLR) 4, the receptor for advanced glycation end products (RAGE), and C-X-C chemokine receptor (CXCR) 4. This study investigated the phenotypes of murine bone-marrow-derived macrophages (BMDMs) stimulated with different HMGB1 redox isoforms using bulk RNA sequencing (RNA-Seq). Disulfide HMGB1 (dsHMGB1)-stimulated BMDMs showed a similar but distinct transcriptomic profile to LPS/IFNγ- and LPS-stimulated BMDMs. Fully reduced HMGB1 (frHMGB1) did not induce any significant transcriptomic change. Interestingly, compared to LPS/IFNγ- and LPS-, dsHMGB1-stimulated BMDMs showed lipid metabolism and foam cell differentiation gene set enrichment, and oil red O staining revealed that both dsHMGB1 and frHMGB1 alleviated oxidized low-density lipoprotein (oxLDL)-induced foam cells formation. Overall, this work, for the first time, used transcriptomic analysis by RNA-Seq to investigate the impact of HMGB1 stimulation on BMDM polarization. Our results demonstrated that dsHMGB1 and frHMGB1 induced distinct BMDM polarization phenotypes compared to LPS/IFNγ- and LPS- induced phenotypes.

## 1. Introduction

Macrophages are myeloid immune cells widely distributed in tissues, playing an important role in inflammation, cell death, and regeneration. Macrophages sense foreign pathogens-derived cues and tissue-derived signals within their microenvironment and respond by initiating innate immune activities and inflammation [1]. Depending on the trigger, macrophages polarize their gene expression towards distinct patterns [1]. Two major macrophage sub-populations with different functional phenotypes that have been recognized are classically activated or inflammatory (M1) and alternatively activated or anti-inflammatory (M2) macrophages [2]. However, macrophage polarization in vivo is a spectrum rather than distinct M1 or M2 phenotypes. There is a growing interest in characterizing the functional phenotypes mediated by immune-activating molecules, such as cytokines and damage-associated molecular patterns (DAMPs), compared to pathogen-associated molecular patterns (PAMPs). 

One such molecule is high-mobility group box 1 (HMGB1), a prototypical DAMP expressed in almost all nucleated cells. During cell homeostasis, HMGB1 is mainly maintained in the nucleus as a DNA-binding protein regulating transcription and genome stability [3]. In inflammatory and injury conditions, HMGB1 is translocated to the cytoplasm and further released to the extracellular space. HMGB1 release occurs from dying cells or activated immune cells, including macrophages [4,5]. The extracellular inflammatory activity of HMGB1 is regulated by the redox state of three cysteine residues at amino acid positions 23, 46 and 106. The three different HMGB1 redox isoforms are denoted: fully reduced HMGB1 (C23HC45HC106H, frHMGB1), disulfide HMGB1 (C23-C45C106H, dsHMGB1, “-” indicating a disulfide bond), and sulfonyl HMGB1 (C23SO_3_C45SO_3_C106SO_3_, oxHMGB1). frHMGB1 is able to form a heterocomplex with C-X-C motif chemokine (CXCL) 12 and bind to C-X-C chemokine receptor (CXCR) 4, inducing cell migration and tissue regeneration [6]. dsHMGB1 is the only HMGB1 isoform known to exhibit a pro-inflammatory role by inducing cytokine release via Toll-like receptor (TLR) 4-MyD88-NFκB signalling pathway [7]. HMGB1 has additionally been reported to interact with a number of receptors, including TLR2, TLR9, and the receptor for advanced glycation end products (RAGE) [8,9,10], which are all expressed by myeloid cells. All redox isoforms of HMGB1 can bind to RAGE [11], while for other HMGB1 receptors, the importance of redox forms has not been defined. 

The pro-inflammatory activity of HMGB1 was first discovered in studies designed to identify novel mediators of sepsis [12]. Today, increased HMGB1 levels have been found in a number of auto-inflammatory and auto-immune diseases, including rheumatoid arthritis [13], systemic juvenile idiopathic arthritis [14], and systemic lupus erythematosus [15]. Additionally, increased levels of HMGB1 have been detected in synovial fluid after acute knee injury [16] and in serum after stroke [17]. The ameliorative effects of HMGB1 neutralization have been demonstrated [18]. However, the roles of the different redox isoforms in different conditions are still not well described due to the lack of a method allowing this distinction. The majority of the studies demonstrating divergent biological activities of HMGB1 redox isoforms are obtained by in vitro studies.

Tissue inflammation is often characterised by an increased number of activated macrophages, and many anti-inflammatory therapies target various macrophage functions. Therefore, it is of relevance to clarify which macrophage functions DAMPs and PAMPs induce and whether there are differences in the induced patterns and outcomes [19,20,21]. We have previously shown that dsHMGB1 and frHMGB1 induced different polarization patterns in murine bone marrow-derived macrophages (BMDMs) [22]. frHMGB1 mainly induced BMDM migration without a distinct polarization signature; dsHMGB1, on the other hand, induced similar but not identical pro-inflammatory mediator secretion patterns as M1. Here, we opted to map the transcriptome profile by RNA sequencing (RNA-Seq) to further define the polarization patterns induced by the prototypic DAMP HMGB1 in its different redox isoforms in comparison to M1 stimulation by LPS and IFNγ or PAMP stimulation by LPS alone. dsHMGB1 and LPS both ligate to the TLR4 complex but on different epitopes in MD2 [7]. Comparing dsHMGB1 and LPS in BMDM polarization would reflect the TLR4-mediated danger signal activated during sterile conditions and during infectious conditions, respectively, and clarify whether a DAMP and a PAMP utilizing the same receptor complex induces an identical response. 

In line with our previous findings, dsHMGB1 triggered an overlapping transcriptome profile to LPS/IFNγ and LPS, while frHMGB1 overlapped with the PBS control, suggesting that frHMGB1 might have limited effects on BMDM polarization. Interestingly, gene set enrichment analysis (GSEA) indicated that dsHMGB1 was involved in lipid metabolism and foam cell differentiation. Compared to the PBS control, both frHMGB1 and dsHMGB1 inhibited oxidized low-density lipoprotein (oxLDL)-induced foam cell formation, while LPS/IFNγ and LPS showed positive effects, revealing a difference between DAMP and PAMP in lipid metabolism of BMDMs. 

## 2. Materials and Methods

### 2.1. Animals

Wild-type female C57BL/6NTac mice (Taconic Biosciences A/S, Lille Skensved, Danmark) were housed in specific pathogen-free facilities at Karolinska University Hospital with free access to water and standard rodent chow. For isolating the bone marrow cells, mice between 8 and 10 weeks of age were euthanized using CO_2_. All of the animal experimental procedures were approved by the Stockholm North Ethical Committee (dnr 18320-2017, N440-12).

### 2.2. Harvesting and Culturing of BMDMs

The bone marrow cells were obtained from femurs as previously described [23]. The cells from mouse hind limbs were matured into macrophages in Dulbecco′s Modified Eagle′s Medium—high glucose (DMEM, Sigma-Aldrich, St. Louis, MO, USA) supplemented with 10% fetal bovine serum (FBS; F7524, Sigma-Aldrich, St. Louis, MO, USA), 2 mM of L-glutamine (Sigma-Aldrich, St. Louis, MO, USA), 1 mM of sodium pyruvate (Sigma-Aldrich, St. Louis, MO, USA), 22 μM of β-mercaptoethanol (Gibco, Paisley, UK), 10,000 I.U./mL of Penicillin–Streptomycin (PenStrep; Sigma-Aldrich, St. Louis, MO, USA), and 10 ng/mL of macrophage colony-stimulating factor (M-CSF; R&D Systems, Minneapolis, MN, USA) for 8–9 days at 37 °C with 5% CO_2_, with half of the medium changing at day 4 and full medium changing at day 6. 

### 2.3. Recombinant HMGB1 Production

Recombinant HMGB1 with a calmodulin-binding protein tag was produced as previously described [24]. To obtain frHMGB1, all of the buffers were supplemented with 5 mM of DTT (Biochemica, Baden-Dättwil, Switzerland). dsHMGB1 (a kind gift from Professor Kevin Tracey’s laboratory, Feinstein Institute, Manhasset, NY, USA) was obtained using buffers without DTT. Endotoxin levels were determined by Limulus assay and were lower than 2.5 EU/mg. The absence of DNA was verified using SDS-PAGE gel electrophoresis and GelRed staining. The purified HMGB1 was stored in PBS ± 0.5 mM DTT. To validate the functionality of dsHMGB1, BMDMs were stimulated with dsHMGB1 at a concentration of 5 µg/mL for 24 h, and significant increases in IL6, IL10, and TNFα secretion from BMDMs were quantified by using commercial ELISA [22]. 

### 2.4. Cell Culture Experiments

The BMDMs were detached from the flask using Trypsin-EDTA (Sigma, St. Louis, MO, USA). The cells were seeded in 24-well culturing plates (Sarstedt, Nümbrecht, Germany) at a density 5 × 10^5^ cells/well in DMEM containing 1% FBS and 10,000 I.U./mL PenStrep and rested overnight. For RNA-Seq, BMDMs from five mice were pooled before seeding; for qPCR verification, BMDMs from each individual mouse were seeded separately. 

To generate cells with an M1 phenotype, 100 ng/mL of LPS-EK Ultrapure (InvivoGen, Toulouse, France) and 20 ng/mL of IFN-γ (R&D Systems, Minneapolis, MN, USA) were added. frHMGB1 and dsHMGB1 were used at a concentration of 5 µg/mL. LPS-EK Ultrapure (InvivoGen, Toulouse, France) was used at a concentration of 1 µg/mL. PBS was included in all experiments as a control. The BMDMs were stimulated 24 h at 37 °C with 5% CO_2_ before RNA isolation. 

### 2.5. RNA-Seq

The total RNA was isolated using an RNeasy Plus Micro Kit (Qiagen, Hilden, Germany) according to the manufacturer’s instructions. RNA was subjected to quality control with Agilent Tapestation according to the manufacturer’s instructions. To construct libraries suitable for Illumina sequencing, the Illumina stranded mRNA prep ligation sample preparation protocol was used with 200 ng of total RNA. The protocol includes mRNA isolation, cDNA synthesis, ligation of adapters, and the amplification of indexed libraries. The yield and quality of the amplified libraries were analyzed using Qubit by ThermoFisher, and the quality was checked by using Agilent Tapestation (Supplementary Table S1). The indexed cDNA libraries were normalized and combined, and the pools were sequenced on the Illumina NextSeq 2000 for a P2 100 cycle sequencing run, generating paired-end reads. Base-calling and demultiplexing were performed using CASAVA software with default settings, generating FASTQ files for further downstream mapping and analysis. The reads were mapped to the GRCm38 reference genome using STAR [25]. Data normalization and the analysis of differential gene expression were conducted using the DESeq2 R-package [26] using a negative binomial test in R studio 4.1.3. Since the RNA samples were from technical replicates rather than biological replicates (i.e., the RNA samples were pooled from five mice), *p*-values from the differential gene expression analysis had to be ignored. Instead, the genes were considered differentially expressed when the Log_2_FoldChange was larger than 2.0 or smaller than −2.0.

The normalized raw counts of the 3952 differentially expressed genes were grouped into modules by using an inverse Pearson correlation as the distance for hierarchical agglomerative clustering with Ward’s method (“ward.D2”), with the defined height cut of the cluster tree equalled to 3.8. Functional gene annotation was performed on each gene module individually, using the Gene Ontology (GO_Biological_Process_2021) libraries with the enrichR R-package [27] in R studio 4.1.3. Gene set enrichment analysis (GSEA) was performed using public software (GSEA 4.1.0) from the Broad Institute [28], with the following settings: the chip platform was “Mouse_Ensembl_Transcript_ID_Human_Orthologs_MSigDB.v7.5.chip”; “collapse” to each probe set in the expression dataset into a single vector for the genes, which is identified by its HGNC gene symbol; “gene_set” permutation type.

### 2.6. Verification of Differentially Expressed Genes Identified in RNA-Seq

The RNA from four individual mice was isolated separately using the RNeasy Plus Micro Kit (Qiagen, Hilden, Germany) and reverse transcripted to cDNA using the iScript cDNA synthesis kit (Bio-Rad, Hercules, CA, USA) according to the manufacturers’ instructions. qPCR was performed using the KiCqStart^®^ SYBR^®^ Green qPCR ReadyMix™ (Sigma, St. Louis, MO, USA) and run on a CFX384 Thermal Cycler according to the manufacturer’s instructions. The primer pairs are specified in Supplementary Table S2. To calculate the ddCt values, the Ct data were normalized against the *Gapdh* reference gene and PBS controls. The data are presented as –ddCt (also called Log_2_FoldChange), i.e., negative values correspond to downregulated gene expression, and positive values correspond to upregulated gene expression in the graphs.

### 2.7. Oil Red O Staining

The BMDMs were stimulated according to Section 2.4 for 2 h before applying 100 μg/mL of oxidized low-density lipoprotein (oxLDL) (Invitrogen, Eugene, OR, USA) for 24 h. BMDMs were then washed with PBS and fixed with 4% paraformaldehyde (Solveco, Sweden) for 15 min at room temperature. The cells were stained with 0.3% (*w*/*v*) Oil Red O (Sigma-Aldrich, St. Louis, MO, USA) in isopropanol (Solveco, Sweden) for 30 min at room temperature, followed by counterstaining with hematoxylin (Sigma-Aldrich, St. Louis, MO, USA) for 30 s. The cells with positive staining were quantified and normalized to hematoxylin counterstaining. 

### 2.8. Statistical Analysis

Statistical analysis was performed using GraphPad Prism version 9.3.1 for Windows (GraphPad Software, San Diego, CA, USA). For the qPCR results, a Shapiro–Wilk test was performed to check the normality of the data. RM-one way ANOVA with Turkey’s multiple comparisons test was performed on the data that were normally distributed; the Friedman test with Dunn’s multiple comparisons test was performed on the data that were not normally distributed. For the foam cell induction assay, statistical comparisons were performed using two-way ANOVA with Šídák’s multiple comparisons. *p*-values below 0.05 were considered statistically significant. 

## 3. Results

### 3.1. dsHMGB1-, LPS- and LPS/IFNγ-Stimulation Resulted in Similar Pro-Inflammatory Gene Expression Patterns, While frHMGB1 Overlapped with PBS Control

**Figure 1:**
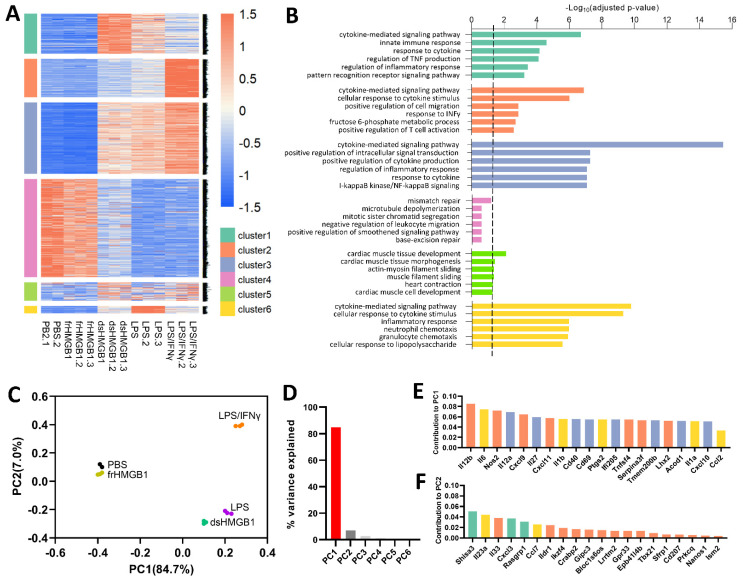
The general overview of transcriptome patterns of BMDMs stimulated 24 h with frHMGB1, dsHMGB1, LPS and LPS/IFNγ. (**A**) Clustered heatmap of normalized counts of 3952 differentially expressed genes. (**B**) Functional annotation of genes in each cluster was condcuted based on Gene Ontology (GO) enrichment. Only the top six enriched gene sets are presented, sorted by adjusted *p*-value. The scatter line indicates the cut-off *p*-value 0.05. (**C**) Principal component analysis (PCA) plot of the samples. (**D**) Percentage of variance explained by principal component (PC) 1 to 6. Only PC1 explained more than 80%; the other PCs were less abundant than PC1; especially none of PC2 to PC6 excessed 10%. (**E**,**F**) Ranking of the top 20 leading genes that contribute to the variance in PC1 and PC2. The colour of the bars of each gene corresponds to the colours of clusters specified in (**A**).

The general overview of the transcriptome profile is presented in Figure 1. Using hierarchical clustering on the differentially expressed genes, we classified the genes into six clusters (Figure 1A). Compared with the PBS control, frHMGB1-stimulation did not result in any significant transcriptomic shift, while dsHMGB1-, LPS- and LPS/IFNγ-stimulation induced similar pro-inflammatory gene expression patterns.

Clusters 1, 2, and 6 represent the genes expressed highest in the dsHMGB1, LPS, and LPS/IFNγ groups, respectively; cluster 3 involves the genes generally higher expressed in dsHMGB1, LPS, and LPS/IFNγ groups compared with the PBS and frHMGB1 groups (Figure 1B). The top GO biological processes of these four clusters commonly include cytokine-mediated signalling pathways (Figure 1B). “Response to IFNγ” in cluster 2 and “cellular response to lipopolysaccharide” in cluster 6 are in agreement with the experimental stimuli (Figure 1B). The genes in cluster 4 generally express lower in the LPS and LPS/IFNγ groups, and the top-enriched GO processes are related to the cell cycle. Interestingly, cluster 5 represents the genes mainly enriched in cardiac muscle development and functioning, though the sequencing was performed with BMDMs.

The PCA plot reveals the three separate clusters: frHMGB1 group clusters close to the PBS control group; dsHMGB1 and LPS groups are close to each other and separate from the PBS control and frHMGB1; the LPS/IFNγ group is approximately equally separated from the other two clusters (Figure 1C). PC1, accounting for more than 84%, is the main variance explaining the separation, and PC2 to PC6 are all below 10% (Figure 1D). The genes that are most accounted for in PC1 are located in clusters 2, 3, and 6 (Figure 1E), while the genes that are most accounted for in PC2 are located in clusters 1, 2, and 6 (Figure 1F); this corresponds to that of dsHMGB1, LPS, and LPS/IFNγ, as the pro-inflammatory groups, are most separated from the control in the PCA plot (Figure 1C). 

### 3.2. Identification of Gene Signatures in dsHMGB1- Compared to LPS/IFNγ-Treated BMDMs

**Figure 2:**
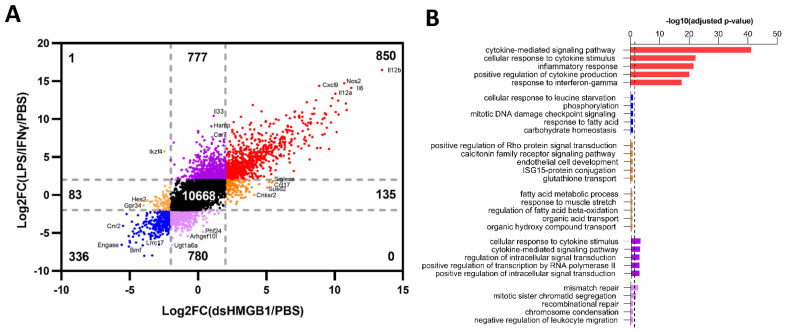
Comparison of common and distinct genes of LPS/IFNγ- and dsHMGB1-stimulated BMDMs. (**A**) Log2FoldChange (Log2FC) vs. Log2FC plot of dsHMGB1 vs. control on the *x*-axis and LPS/IFNγ vs. control on the *y*-axis highlighting common upregulated genes in red (850 genes), common downregulated genes in blue (336 genes), LPS/IFNγ up- and dsHMGB1 downregulated genes in dark yellow (1 gene), dsHMGB1-distinct genes in orange (218 genes) and LPS/IFNγ-distinct genes (1557 genes) in purple. Grey dotted lines represent a ±2 cut-off. (**B**) GO analysis on six gene sets, only the top five enriched biological processes are presented, sorted by adjusted *p*-value. The scatter line indicates the cut-off *p*-value 0.05.

We have previously demonstrated that dsHMGB1 induced BMDM polarization towards a pro-inflammatory phenotype that differed from the classical M1, induced by LPS/IFNγ, regarding migratory abilities and nitric oxide secretion [22]. To dissect the gene signatures of LPS/IFNγ- and dsHMGB1-stimulated BMDMs, and to explore the potential transcriptional explanations for our previous findings, the gene expression values were plotted into a FoldChange (FC) vs. FC plot (Figure 2A). Using the 2 and −2 Log2FoldChange lines as a reference, we identified 850 co-upregulated and 336 co-downregulated genes between LPS/IFNγ- and dsHMGB1-treated BMDMs. Overall, 135 genes were distinctively upregulated, and 83 genes were distinctively downregulated by dsHMGB1, and these two gene sets were regarded as “dsHMGB1-distinct”. Furthermore, 777 genes were distinctively upregulated, and 780 genes were distinctively downregulated by LPS/IFNγ, and these two gene sets were regarded as “LPS/IFNγ-distinct”.

To outline the enriched biological processes of the six gene sets, we undertook GO enrichment analysis on each gene set (Figure 2B). Co-upregulated gene set resulted in the most significant enrichment, and the top-ranked biological processes were “cytokine-mediated signaling pathway”, “cellular response to cytokine stimulus”, “inflammatory response”, “positive regulation of cytokine production”, and “response to interferon-gamma”. The co-downregulated gene sets and dsHMGB1-distinct gene sets did not show significant enrichment. LPS/IFNγ-distinct upregulated gene set showed similar inflammation-related enrichment as the co-upregulated gene set, while LPS/IFNγ-distinct downregulated gene set only had two significantly enriched biological processes, named “mismatch repair” and “mitotic sister chromatid segregation”, these results were in concordance with Figure 1A. Our data demonstrate that the DAMP molecule HMGB1, in its disulfide isoform, induces a gene expression pattern distinct from but similar to the classical M1 stimulated by LPS/IFNγ.

**Figure 3:**
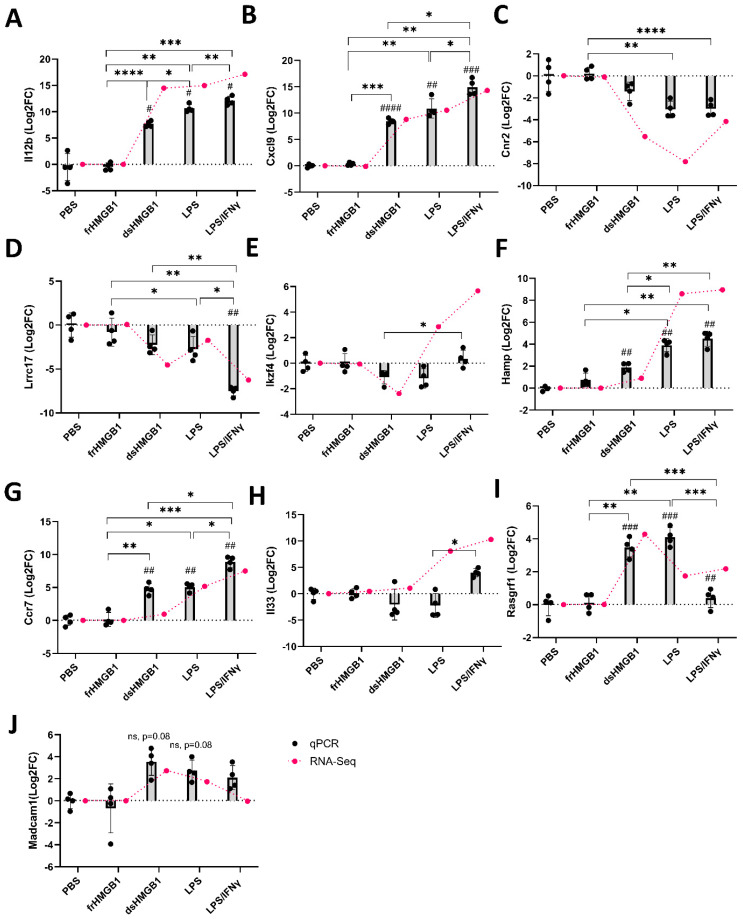
qPCR validation of the featured genes identified by RNA-Seq. Expression of *Il12b* (**A**), *Cxcl9* (**B**), *Cnr2* (**C**), *Lrrc17* (**D**), *Ikzf4* (**E**), *Hamp* (**F**), *Ccr17* (**G**), *Il33* (**H**), *Rasgrf1* (**I**), *Madcam1* (**J**) were determined using qPCR in BMDMs stimulated for 24 h by frHMGB1, dsHMGB1, LPS and LPS/IFNγ (M1), *n* = 4 mice, 2 replicates/mouse/condition. Gene expression is represented as Log2FoldChange (Log2FC) relative to the mean of the PBS group, and the scale bars represent the standard deviations (SD). Red dots represent the RNA-Seq results. Statistical comparisons were performed using RM-one way ANOVA with Turkey’s multiple comparisons test on data that are normally distributed or using Friedman test with Dunn’s multiple comparisons on data that are not normally distributed. # = significant comparing with PBS control; * = significant comparing between two treatment groups. */#, *p* < 0.05; **/##, *p* < 0.01; ***/###, *p* < 0.001; ****/####, *p* < 0.0001.

To validate the RNA-Seq data, qPCR was performed on a selected set of genes: *Il12b* and *Cxcl9* (Figure 3A,B) among the co-upregulated genes; *Cnr2* and *Lrrc17* among the co-downregulated genes (Figure 3C,D); *Ikzf4*, upregulated in LPS/IFNγ- but downregulated in dsHMGB1-stimulated BMDMs (Figure 3E); *Hamp* (Figure 3F), *Ccr7* (Figure 3G) and *Il33* (Figure 3H) were significantly upregulated in LPS/IFNγ- than dsHMGB1-stimulated BMDMs. Similar gene expression patterns were demonstrated by both RNA-Seq and qPCR. 

### 3.3. Identification of Gene Signatures in dsHMGB1- Compared to LPS-Treated BMDMs

The presence of PAMPs or DAMPs during infection and tissue injury promotes macrophage activation and induces inflammation. Both dsHMGB1 and LPS are ligands for TLR4; however, dsHMGB1 is an endogenous molecule, whereas LPS is a bacterial component. We performed additional experiments to understand if TLR4 ligands could signal differently and whether it is of bacterial origin and initiates an inflammatory response suitable for combatting infection or as an alarmin indicating tissue trauma. 

**Figure 4:**
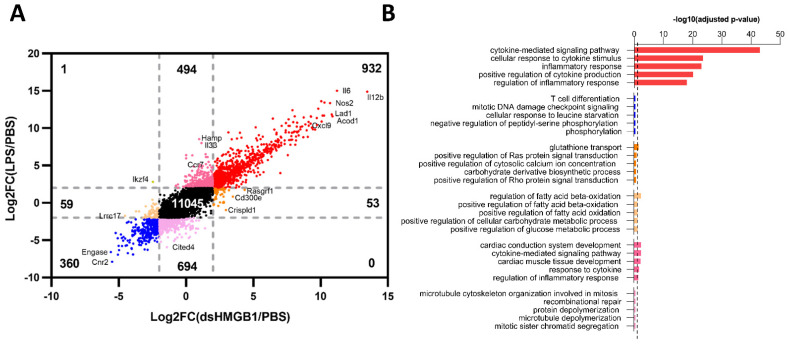
Comparison of common and distinct genes of LPS- and dsHMGB1-stimulated BMDMs. (**A**) Log2FoldChange (Log2FC) vs. Log2FC plot of dsHMGB1 vs. control on the *x*-axis and LPS vs. control on the *y*-axis highlighting common upregulated genes in red (932 genes), common downregulated genes in blue (360 genes), LPS up- and dsHMGB1 downregulated genes in dark yellow (1 gene), dsHMGB1-distinct genes in orange (112 genes) and M1-distinct genes (1188 genes) in pink. Grey dotted lines represent a ±2 cut-off. (**B**) GO analysis on six gene sets; only the top five enriched biological processes are presented, sorted by *p*-value. The scatter line indicates the cut-off *p*-value 0.05.

Using the 2 and −2 Log2FoldChange lines as a reference, we identified 920 co-upregulated and 360 co-downregulated genes between LPS- and dsHMGB1-stimulated BMDMs (Figure 4A). There were 53 genes that were distinctively upregulated, and 59 genes were distinctively downregulated by dsHMGB1, and these two gene sets were regarded as “dsHMGB1-distinct”. There were 494 genes that were distinctively upregulated, and 694 genes were distinctively downregulated by LPS, and these two gene sets were regarded as “LPS-distinct” (Figure 4A). GO analysis of dsHMGB1-distinct areas revealed that the dsHMGB1-upregulated genes were significantly enriched in the “glutathione transport” process, while dsHMGB1-downregulated genes were significantly enriched in biological processes related to fatty acid oxidation (Figure 4B). Similar to LPS/IFNγ-distinct genes, LPS-distinct upregulated gene set showed inflammation-related enrichment (Figure 4B), indicating PAMPs induced stronger pro-inflammatory effects than endogenous molecules.

The genes with altered expression levels specific for dsHMGB1 stimulation are listed in Supplementary Figure S1. In comparison with both LPS- and LPS/IFNγ-stimulation, there were 34 genes distinctively upregulated and 27 genes distinctively downregulated by dsHMGB1 stimulation. To confirm the gene expression differences discovered by RNA-Seq (Figure 2 and Figure 4), the selected genes were verified by qPCR. Compared with the RNA-Seq results, *Rasgrf1* and *Madcam1* were similarly expressed in dsHMGB1-stimulated BMDMs by qPCR verification (Figure 3I,J). However, these two genes were also upregulated with Log2FC larger than 2.0 in the LPS group, which was contradictory to the RNA-Seq data.

To sum up, our results indicated that dsHMGB1 and LPS, both known as TLR4 ligands but of different origin, induced distinct but similar gene expression patterns in BMDMs. The difference recorded were less pronounced than those recorded when comparing dsHMGB1- and LPS/IFNγ-stimulated BMDMs. 

### 3.4. frHMGB1 and dsHMGB1 Attenuated BMDM-Derived Foam Cell Formation

**Figure 5:**
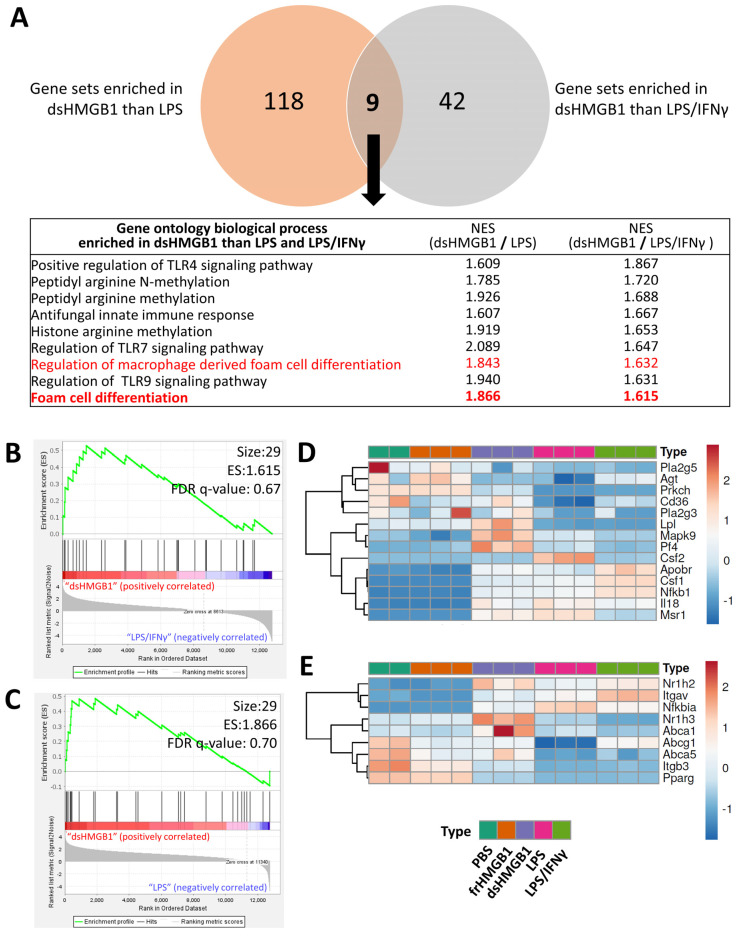
Foam cell differentiation-related gene sets were enriched by dsHMGB1 than LPS and LPS/IFNγ. (**A**) GSEA analysis revealed that 127 gene sets were enriched by dsHMGB1 than LPS, 51 gene sets were enriched by dsHMGB1 than LPS/IFNγ (NES cut-off = 1.6). Nine sets were commonly enriched, among which two were related to foam cell differentiation. (**B**) Foam cell differentiation was enriched by dsHMGB1 (left, red) comparing with LPS/IFNγ (right, blue). (**C**) Foam cell differentiation was enriched by dsHMGB1 (left, red) comparing with LPS (right, blue). (**D,E**) The genes included in positive (D) and negative (E) regulation of macrophage-derived foam cell differentiation (GO:0010744 and GO:0010745) were listed and heatmap which was created based on the normalized gene counts in each sample. Colour intensity was scaled within each row so that the highest value corresponds to red and the lowest to blue. Abbreviation: ES, enrichment score; NES, normalized enrichment score; FDR, False Discovery Rate.

To further identify the biological process enriched by dsHMGB1 compared to LPS and LPS/IFNγ, GSEA was performed comparing dsHMGB1 with LPS and dsHMGB1 with LPS/IFNγ groups. There were 127 and 51 gene sets enriched in the dsHMGB1 group compared to the LPS and LPS/IFNγ groups, respectively, and nine gene sets were commonly enriched in both comparisons (Figure 5A). The gene sets related to TLR4, TLR7, and TLR9 signalling and related to protein methylation, which is important in epigenetic modifications, were enriched in the dsHMGB1 group. Interestingly, two of the nine commonly enriched gene sets were related to foam cell differentiation. The enrichment plot showed that dsHMGB1 resulted in foam cell differentiation gene set enrichment compared to the LPS/IFNγ (Figure 5B) and LPS (Figure 5C) groups.

Foam cell, which forms through dysregulated lipid metabolism, is a subtype of macrophage. As we aimed to characterize the special macrophage phenotype induced by HMGB1 stimulation, we proceeded to further investigate whether HMGB1 induced foam cell differentiation. The genes related to foam cell differentiation were separated into positive regulation (GO:0010744, listed in Figure 5D) and negative regulation (GO:0010745, listed in Figure 5E) groups. However, we were not able to define dsHMGB1 as a positive or negative regulator of foam cell formation: dsHMGB1 resulted in partial gene upregulation and partial gene downregulation compared with the PBS control. The expression of the selected genes (*Lpl* and *Pf4* from the positive list and *Nr1h3* and *Abca1* from the negative list) were quantified by qPCR (Supplementary Figure S2). However, the qPCR results were not in line with the RNA-Seq data: only *Nr1h3* was upregulated by dsHMGB1 than frHMGB1 with a relatively small fold change, while the other three genes were downregulated by dsHMGB1 (Supplementary Figure S2).

**Figure 6:**
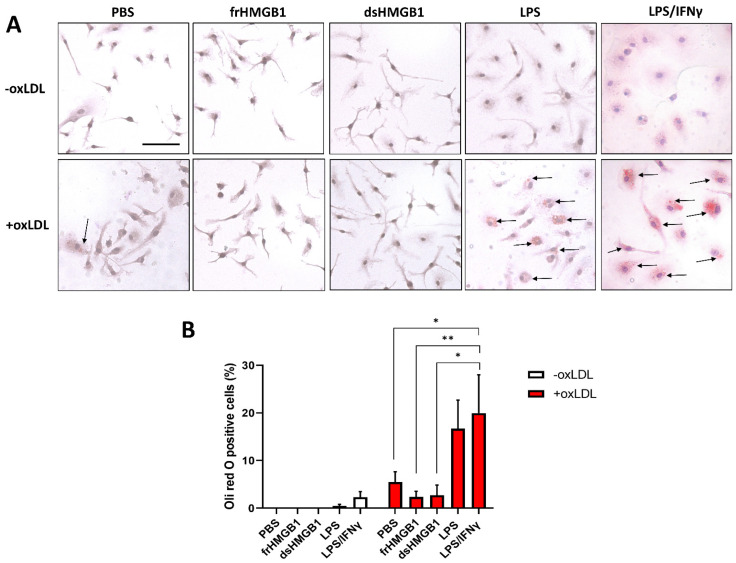
Effects of HMGB1 on foam cell formation from BMDMs. After incubation with frHMGB1 (5 µg/mL), dsHMGB1 (5 µg/mL), LPS (1 µg/mL) or LPS/IFNγ (100 ng/mL LPS and 20 ng/mL IFNγ) for 2 h, BMDMs were treated with oxLDL (100 μg/mL) for 24 h. (**A**) Oil red O staining in stimulated BMDMs. Scale bar = 10 μm. (**B**) The percentage of cells with lipid droplets stained using oil red O in BMDMs with and without oxLDL induction, normalized with haematoxylin counterstaining. Average was calculated from the three pictures taken from each cell culture well, *n* = 4 mice, mean of 4+SEM. Statistical comparisons were performed using two-way ANOVA with Šídák’s multiple comparisons, *, *p* < 0.05; **, *p* < 0.01.

To further explore the GSEA suggested involvement of dsHMGB1 in foam cell differentiation, we stimulated BMDMs with oxLDL, a commonly used molecule to induce in vitro foam cell formation. Stimulated-BMDMs were stained with oil red O to detect neutral lipids and lipid droplets. After 24-h incubation, there was almost no oil red positive staining in frHMGB1- or dsHMGB1-stimulated BMDMs, regardless of oxLDL stimulation; in contrast, lipid droplet accumulation was observed in PBS, LPS, and LPS/IFNγ groups after oxLDL stimulation (Figure 6A). Quantification of positive staining demonstrated an equal oxLDL uptake in M1- and LPS-stimulated BMDMs and a lower uptake in control BMDMs (Figure 6B). 

We continued to investigate whether increasing HMGB1 concentration influenced lipid metabolism. Up to 50 µg/mL, both frHMGB1 and dsHMGB1 stimulation resulted in an elongated cell shape (Supplementary Figure S3B); dsHMGB1 showed cytotoxicity at 100 µg/mL, while frHMGB1 was not cytotoxic in any of the tested concentrations (Supplementary Figure S3A). By increasing the concentration, both dsHMGB1 and frHMGB1 led to some lipid droplet accumulation after oxLDL stimulation, but not more than the PBS control (Supplementary Figure S3B,C). In summary, we could not confirm that the suggested feature of dsHMGB1 induces BMDM-derived foam cell formation, and neither did the frHMGB1 investigated in parallel.

## 4. Discussion

Macrophages play important roles in both tissue homeostasis and during inflammatory conditions. Macrophages are plastic, and their functional subtypes develop in response to triggering cues in the local environment. A deepened understanding of the transcriptional profiles and the molecular pathways associated with the different macrophage subtypes is essential for the further characterisation of the complex biology of macrophages. Macrophage polarization, the differentiation into functional phenotypes, is affected by both exogenous triggers, i.e., PAMPs, and endogenous triggers, i.e., DAMPs. In this study, we report the impact on macrophage polarization of the prototypic DAMP molecule HMGB1. This is, to our knowledge, the first study reporting gene expression profiles induced by different redox isoforms of HMGB1 on BMDMs. 

We used simultaneous stimulations with both LPS and IFNγ to induce M1 cells, which is the standard protocol [29]. Using RNA-Seq, we corroborated our previously reported findings that dsHMGB1 resulted in a distinct but similar gene expression profile to LPS/IFNγ-stimulated BMDMs. Overall, 218 genes were either up- or downregulated by dsHMGB1 but unchanged by LPS/IFNγ stimulation. However, no significant GO enrichment could be defined based on these genes. Overall, 1186 genes were either up- or downregulated by both dsHMGB1 and LPS/IFNγ stimulation, and these genes showed enrichment in inflammatory processes, as well as cytokine response and regulation. These data provide a molecular explanation for the earlier assumption that dsHMGB1 induces an M1-like phenotype. 

We then compared the gene expression patterns after stimulation with dsHMGB1 and LPS alone. The comparison revealed highly similar transcriptomic changes with only 112 genes specifically up- or downregulated by dsHMGB1. Again, no specific significant GO enrichment could be defined. This was expected as dsHMGB1 and LPS both ligate to TLR4 complex, and both have been previously demonstrated to activate the NFκB signalling pathway [7,30]. In this study, we demonstrated that TLR4 activation by the investigated PAMP and DAMP resulted in highly similar transcriptomic changes. 

To validate the RNA-Seq findings, we chose to quantify gene expression by qPCR for a selected set of genes. Overall, there was a good correspondence between RNA-Seq and qPCR results. *Rasgrf1* and *Madcam1* were higher expressed in the dsHMGB1-stimulated than LPS/IFNγ-stimulated BMDMs. *Rasgrf1* encodes the protein named Ras guanine nucleotide releasing factor 1 (RasGRF1), which was detected in synovial macrophages in situ [31]. RasGRF1 was significantly enhanced in rheumatoid arthritis synovial tissue, and it contributed to Matrix metalloproteinase-3 (MMP-3) production [31]. Mucosal Addressin Cell Adhesion Molecule 1 (MAdCAM1) has been demonstrated to regulate lymphocyte migration [32] and immune cell infiltration [33]. Our data thus suggest mechanisms by which dsHMGB1 can contribute to joint destruction and cell migration. *Hamp*, encoding the protein Hepcidin, was higher expressed in both LPS- and LPS/IFNγ-stimulated than dsHMGB1-stimulated BMDMs. Hepcidin is a regulator of iron metabolism. Increased hepcidin expression results in the retention of iron in macrophages, leading to oxidative stress [34,35]. This may explain our previous finding that nitric oxide (NO) was only measurable from LPS/IFNγ-stimulated but not dsHMGB1-stimulated BMDMs [22]. Again, these results clearly indicated the dsHMGB1 induced a phenotype different from the classical M1.

We used GSEA to further study potential enrichments in biological processes. In GSEA, the enrichment of a defined set of genes, previously associated with a certain pathway or process, is analyzed for enrichment. Overall, 127 gene sets were enriched by dsHMGB1 as compared to LPS, and 51 gene sets were enriched by dsHMGB1 as compared to LPS/IFNγ. Of these, nine gene sets were shared and contained three TLR regulatory processes, for TLR4 7 and 9; three protein methylation processes, one anti-fungal process and two foam cell-associated processes. The TLR4 process supports our data on dsHMGB1 inducing a distinct macrophage phenotype. TLR7 and TLR9 are in line with the previously described role of HMGB1 in endosomal uptake of LPS via RAGE for further translocation to cytosolic LPS receptors [36]. HMGB1 could play a similar role in the uptake of TLR7 and TLR9 ligands for deposit in endosomes expressing TLR7 and TLR9. Protein methylation are processes important for epigenetic regulation of the chromatin [37]. How HMGB1 participates in such processes deserves further studies, as does the potential role of HMGB1 in anti-fungal immunity.

Foam cells in atherosclerotic lesions mainly originate from either circulated monocyte-derived macrophages or smooth muscle cells [38,39,40]. Foam cells are formed through dysregulated lipid metabolism resulting in intracellular storage of lipid droplets [41]. HMGB1 has been reported to promote cholesterol accumulation in vascular smooth muscle cells [42] and to accelerate oxLDL-induced foam cell formation and apoptosis in RAW264.7 cells [43]. Thus, to test whether dsHMGB1 polarized BMDMs to foam cells, we stimulated BMDMs with fr- and dsHMGB1 and subsequently fed the cells oxLDL. In contrast to the studies above, our data revealed that both dsHMGB1- and frHMGB1-stimulation restrained foam cell formation. 

OxLDL is taken by the cells via the scavenger receptors. We found that scavenger receptors (*Scarb1*, *Scarb2*, *Cd36*, *Colec12*, *Scara3*, *Cd68*, and *Olr1*) binding oxLDL were differentially expressed among the five groups (Supplementary Figure S4). LPS/IFNγ-stimulated BMDMs showed higher expression of *Olr1*, which encodes the oxLDL receptor lectin-like oxidized low-density lipoprotein receptor-1 (LOX-1). LOX-1 is upregulated after exposure to pro-inflammatory and pro-atherogenic stimuli and can be detected in atherosclerotic lesions [44]. Lee et al. [45] demonstrated that HMGB1 enhanced oxLDL uptake through induction of LOX-1 in human coronary artery endothelial cells, while our RNA-Seq results or in vitro foam cell induction experiments were not in line with this finding. One potential explanation could be that HMGB1 acts as an antagonist of oxLDL receptors, and the pre-treatment of HMGB1, therefore, inhibited oxLDL uptake. However, there has been no study investigating the binding between HMGB1 and the scavenger receptors of oxLDL in BMDMs. Future confirmation studies are highly warranted.

HMGB1-stimulated BMDMs showed a stretched and elongated shape, which we have previously observed in a scratch assay, where both fr- and dsHMGB1 induced BMDM migration [22]. The function data can be explained by RNA-Seq data indicating that LPS/IFNγ-stimulated BMDMs seemed to show an impairment of the cell cycle. Earlier studies suggested that M1 mounted a rapid and effective immune response against highly proliferative intracellular pathogens, producing NO, reactive oxygen species (ROS), and pro-inflammatory cytokines. This process is rapid and energy-intensive, causing macrophages to exit from the cell cycle during M1 differentiation, indicating potential coordination between metabolic regulation and macrophage physiology [46,47]. 

Our study has some limitations. Firstly, the RNA-Seq was only performed on technical replicates, and a few of the RNA-Seq results were not reproducible by qPCR verification. This could be due to either the biological variation or the different quantification principles between RNA-Seq and qPCR, for example, sequencing depth and the specific transcripts measured. Additionally, oxHMGB1 was not included in this study. Although there has been no inflammation-generation function reported, oxHMGB1 was found to be an anti-inflammatory molecule resulting in immunocompetent cell recruitment to inhibit cytotoxic cells [48]. Furthermore, we studied gene expression occurring after 24 h; the expression changes occurring in earlier or later time points were not investigated. For instance, we previously identified the *Il6* expression peak after dsHMGB1 stimulation for 7 h [22]. 

Taken together, our data indicated that though dsHMGB1 resulted in M1-like pro-inflammatory cytokine release and a pro-inflammatory gene expression phenotype, the elongated cell shape and reduced foam cell development feature were closer to the M2-like anti-inflammatory phenotype.

## 5. Conclusions

We report for the first time the effect of different HMGB1 redox forms on macrophage polarization by using RNA-Seq. Our results revealed that frHMGB1 did not induce any significant transcriptomic changes compared to PBS control. dsHMGB1 induced a pro-inflammatory phenotype, which confirmed current knowledge and gave a more in-depth understanding of the downstream effects. Interestingly, dsHMGB1 induced similar transcriptomic changes as LPS but differed more from the classical M1. As opposed to M1, dsHMGB1-stimulated macrophages retained their migratory capacity. It is interesting to speculate that dsHMGB1, as a DAMP, induces a macrophage phenotype on the M1 end of the polarization spectrum though more fine-tuned to sterile inflammatory conditions, to deal with cell death, tissue injury and subsequent regeneration rather than defence against pathogens, evident by its suggested role in TLR7 and TLR9 regulation and retained migratory capacity. Finally, our study also suggested that both frHMGB1 and dsHMGB1 attenuated foam cell formation from BMDMs.

## Data Availability

All the RNA-Seq raw data generated in this study were deposited at the Gene Expression Omnibus under accession number GSE200210. Other data would be available on request from the corresponding author.

## Supplementary Materials

The following supporting information can be downloaded at: https://www.mdpi.com/article/10.3390/biom12060779/s1, Supplementary Table S1. RNA-Seq quality control, Supplementary Table S2. qPCR primer pairs sequence, Supplementary Figure S1. dsHMGB1-stimulation distinctively upregulated and downregulated genes comparing with LPS- and LPS/IFNγ-stimulation, Supplementary Figure S2. qPCR validation of the foam cell formation featured genes identified by RNA-Seq, Supplementary Figure S3. Effects of HMGB1 on foam cell formation from BMDMs, Supplementary Figure S4. Gene expression of scavenger receptors to oxLDL.
