# Supplementary material for: Transcriptomic Profiling Reveals That HMGB1 Induces Macrophage Polarization Different from Classical M1
Source: Biomolecules. 2022 Jun 2;12(6):779. doi: 10.3390/biom12060779 (PMC9221381; doi:10.3390/biom12060779)
Supplement: Supplementary file 1 [file biomolecules-12-00779-s001.zip › biomolecules-1749249-supplementary.pdf]

Transcriptomic Profiling Reveals that HMGB1 Induces Macrophage Polarization Different from Classical M1

Supplementary documents

Contents

Supplementary Tables 2

Supplementary Table S1. RNA-Seq quality control 2

Supplementary Table S2. qPCR primer pairs sequence 2

Supplementary Figures 3

Supplementary Figure S1. dsHMGB1-stimulation distinctively upregulated and downregulated genes comparing with LPS- and LPS/IFNγ-stimulation 3

Supplementary Figure S2. qPCR validation of the foam cell formation featured genes identified by RNA-Seq 4

Supplementary Figure S3. Effect of HMGB1 on foam cell formation from BMDM. 5

Supplementary Figure S4. Gene expression of scavenger receptors to oxLDL.. 6

Supplementary Tables

Supplementary Table S1. RNA-Seq quality control

Sample	Barcode sequence	PF Clusters	% of the lane	% Perfect barcode	% One mismatch barcode	Yield (Mbases)	% PF Clusters	% >= Q30 bases	Mean Quality Score
PBS-1	TGGTACCTAA+AGTACTCATG	40,219,989	10.31	97.29	2.71	4,183	100	91.3	32.67
PBS-2	TTGGAATTCC+GTATTGACGT	40,959,722	10.5	96.97	3.03	4,260	100	91.34	32.67
PBS-3	CCTCTACATG+AGGAGGTATC	32,074,721	8.22	97.69	2.31	3,336	100	91.1	32.63
M1-1	GGAGCGTGTA+ACTTACGGAT	41,671,277	10.68	98.06	1.94	4,334	100	91.19	32.65
M1-2	GTCCGTAAGC+AAGATACACG	41,829,791	10.72	97.7	2.3	4,350	100	91.26	32.66
M1-3	ACTTCAAGCG+TTCATGGTTC	49,042,165	12.57	96.92	3.08	5,100	100	91.05	32.62
dsHMGB1-1	TCAGAAGGCG+TATGATGGCC	40,424,779	10.36	89.65	10.35	4,204	100	90.96	32.61
dsHMGB1-2	GCGTTGGTAT+GGAAGTATGT	48,463,863	12.42	97.23	2.77	5,040	100	91.41	32.69
dsHMGB1-3	ACATATCCAG+ATTGCACATA	41,914,272	10.75	96.97	3.03	4,359	100	91.2	32.65
LPS-1	GAGACGAT+ACCGGTTA	35,088,196	7.83	97.06	2.94	4,281	100	93.78	33.06
LPS-2	CTTGTCGA+CGATGTTC	30,944,204	6.9	97.71	2.29	3,775	100	93.76	33.06
LPS-3	TTCCAAGG+CTACAAGG	31,647,602	7.06	96.95	3.05	3,861	100	93.81	33.07
frHMGB1-1	CTGATCGT+GCGCATAT	26,770,027	5.97	96.65	3.35	3,266	100	93.65	33.04
frHMGB1-2	ACTCTCGA+CTGTACCA	27,519,820	6.14	97.48	2.52	3,357	100	93.46	33
frHMGB1-3	TGAGCTAG+GAACGGTT	26,044,916	5.81	95.87	4.13	3,177	100	93.98	33.1

Supplementary Table S2. qPCR primer pairs sequence

Gene	F/R	Sequence(5'-3')
Abca1	F	GGTCTCCAGAAGGTATTTTTG
	R	TCAGGATGTCCATGTTGTAG
Ccr7	F	GAGAGAGACAAGAACCAAAAG
	R	CTGGAAAATGACAAGGAGAG
Cnr2	F	AAAGTGTGAGAGCAAGAAAC
	R	TTTGGCTTCTTCTACTGGAG
Cxcl9	F	GAGGAACCCTAGTGATAAGG
	R	GTTTGATCTCCGTTCTTCAG
Gapdh	F	CAAGGTCATCCATGACAACTTTG
	R	GTCCACCACCCTGTTGCTGTAG
Hamp	F	CATCAACAGATGAGACAGAC
	R	ATTTACAGCAGAAGATGCAG
Il12b	F	CATCAGGGACATCATCAAAC
	R	CTCTGTCTCCTTCATCTTTTC
Il33	F	GAACATGAGTCCCATCAAAG
	R	CAGCTGGTTATCTTTTACTCC
Lpl	F	GAGACTCAGAAAAAGGTCATC
	R	GTCTTCAAAGAACTCAGATGC
Lrrc17	F	GAGGAAAGAGTTGAAGAAAGTC
	R	GTGTATGAGCCCTAAAAAGG
Madcam1	F	GGAGATTCCAGTACTACAGAG
	R	TTGATGAGGTCAGGATGTAG
Nr1h3	F	GATGTTTCTCCTGATTCTGC
	R	CTCCAACCCTATCCCTAAAG
Osbpl3	F	CAAGAGCCAAGCTGATATTG
	R	GTTCTTCTGACTTCACCTTC
Pf4	F	TAGCCACCCTGAAGAATG
	R	GACATTTAGGCAGCTGATAC

Supplementary Figures

A

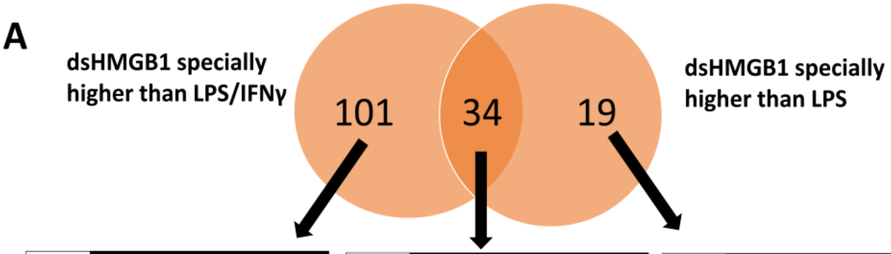

	Log2FC (dsHMGB1/ control)	Log2FC (LPS/IFNγ/ control)	Log2FC (LPS/ control)
Siglece	5.49	1.85	3.75
Ccl17	5.41	1.65	9.05
Dscaml1	5.20	1.73	5.95
Susd2	5.07	0.99	4.07
Sh2d4a	4.48	1.45	3.70
Cnksr2	4.12	0.00	2.50
Spib	3.94	1.01	2.16
C1qtnf7	3.77	1.89	2.93
Ppm1n	3.75	0.41	2.37
Gdf15	3.73	1.43	4.41

	Log2FC (dsHMGB1/ control)	Log2FC (LPS/IFNγ/ control)	Log2FC (LPS/ control)
Cd300e	3.48	-0.28	0.84
Aoc1	3.19	0.00	1.69
Crispld1	2.96	1.30	-0.96
Tshz1	2.90	1.01	1.82
Osbpl3	2.85	-0.88	1.45
Dnmt3l	2.80	-1.17	0.13
Slc13a3	2.79	1.22	0.84
Madcam1	2.77	0.02	1.70
Fam72a	2.76	1.97	1.97
Clec4a1	2.64	0.59	1.83

	Log2FC (dsHMGB1/ control)	Log2FC (LPS/IFNγ/ control)	Log2FC (LPS/ control)
Rasgrf1	4.36	2.32	1.74
Mir99ahg	2.86	2.05	1.82
Apol7b	2.81	4.06	0.00
Myl2	2.69	3.77	1.20
B3gnt5	2.59	2.50	1.75
Mtfr2	2.36	2.29	1.41
Tnfrsf13c	2.36	5.70	1.69
Art2a	2.33	2.02	1.47
Kcna4	2.24	3.75	0.53
Hs3st3b1	2.23	3.55	1.87

B

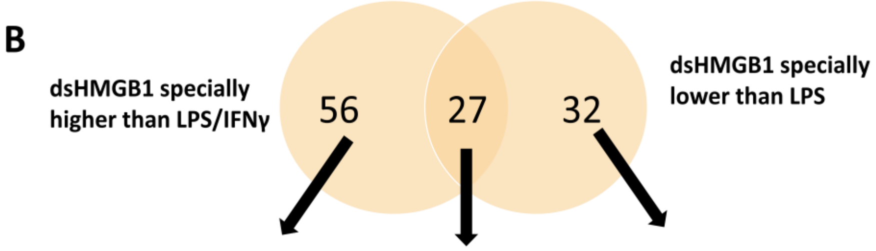

	Log2FC (dsHMGB1/ control)	Log2FC (LPS/IFNγ/ control)	Log2FC (LPS/ control)
Gpr34	-3.99	-1.37	-3.82
Clec9a	-3.52	-0.92	-3.37
Bbs12	-3.37	-0.85	-3.65
Tmem154	-3.23	-1.24	-2.71
Ephb3	-3.21	-1.98	-4.42
Dna2	-3.18	-1.40	-3.99
C5ar2	-3.13	-0.22	-2.21
Sstr2	-3.11	-0.70	-2.26
Slc7a14	-2.95	-0.86	-4.46
Mirt1	-2.92	-1.98	-2.57

	Log2FC (dsHMGB1/ control)	Log2FC (LPS/IFNγ/ control)	Log2FC (LPS/ control)
Hes2	-3.72	-0.71	-1.13
Ackr4	-3.05	-1.49	-1.88
Plekhh2	-3.04	-0.32	-1.60
Adh7	-3.00	-1.21	-0.46
Zfp960	-2.74	-1.24	-1.21
Clmn	-2.73	-0.67	-1.79
Fxyd7	-2.58	0.40	-1.45
Kif27	-2.56	0.04	-0.90
Fgf5	-2.55	-0.95	-0.44
Ccdc89	-2.51	-0.45	-1.28

	Log2FC (dsHMGB1/ control)	Log2FC (LPS/IFNγ/ control)	Log2FC (LPS/ control)
Lrrc17	-4.54	-6.26	-1.73
Slc9a2	-3.49	-2.24	-1.13
Sertad4	-3.17	-3.30	-1.98
Depdc1b	-3.01	-2.39	-1.93
Prkag2os2	-2.96	-2.34	-1.80
Lmod1	-2.86	-2.32	-1.85
Nim1k	-2.79	-2.92	-1.50
Ccdc30	-2.70	-2.07	-1.08
Dzank1	-2.64	-2.45	-1.53
Cdh10	-2.62	-2.02	-1.94

Supplementary Figure S1. dsHMGB1-stimulation distinctively upregulated and downregulated genes comparing with LPS- and LPS/IFNγ-stimulation. (A) 135 genes were upregulated by dsHMGB1 but not significantly changed by LPS/IFNγ; 53 genes were upregulated by dsHMGB1 but not significantly changed in LPS. There were 34 common genes between the two lists. The top 10 genes of each compart with their log2FoldChange (Log2FC) in dsHMGB1, LPS/IFNγ, LPS groups are listed below the Venn diagram. Genes highlighted in red were validated by qPCR in Figure 4. (B) 83 genes were upregulated by dsHMGB1 but not significantly changed by LPS/IFNγ; 59 genes were upregulated by dsHMGB1 but not significantly changed in LPS. There were 27 common genes between the two lists. The top 10 genes with their log2FC in dsHMGB1, LPS/IFNγ, LPS groups are listed below the Venn diagram.

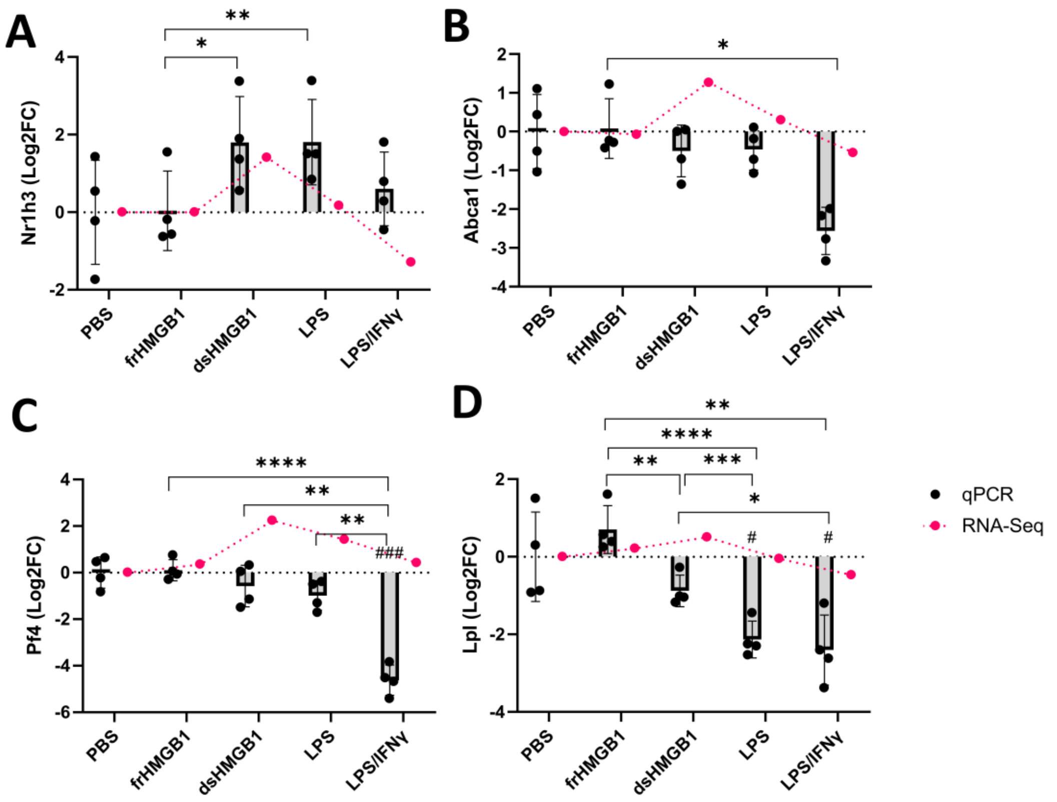

Supplementary Figure S2. qPCR validation of the foam cell formation featured genes identified by RNA-Seq. Expression of *Nr1h3* (A), *Abca1* (B), *Pf4* (C) and *Lpl* (D) were determined using qPCR in BMDMs stimulated for 24 hours by frHMGB1, dsHMGB1, LPS and LPS/IFNγ, n=4 mice, 2 replicates/mouse/condition. Gene expression is represented as Log2FoldChange (Log2FC) relative to the mean of the PBS group, and the scale bars represent the standard deviations. Red dots represented the RNA-Seq results. Statistical comparisons were performed using RM-one way ANOVA with Turkey's multiple comparisons test on data that are normally distributed or using Friedman test with Dunn's multiple comparisons on data that are not normally distributed. # = significant comparing with control; *=significant comparing between two treatment groups. */#, p< 0.05; **, p< 0.01; ***/###, p< 0.001; ****, p< 0.0001.

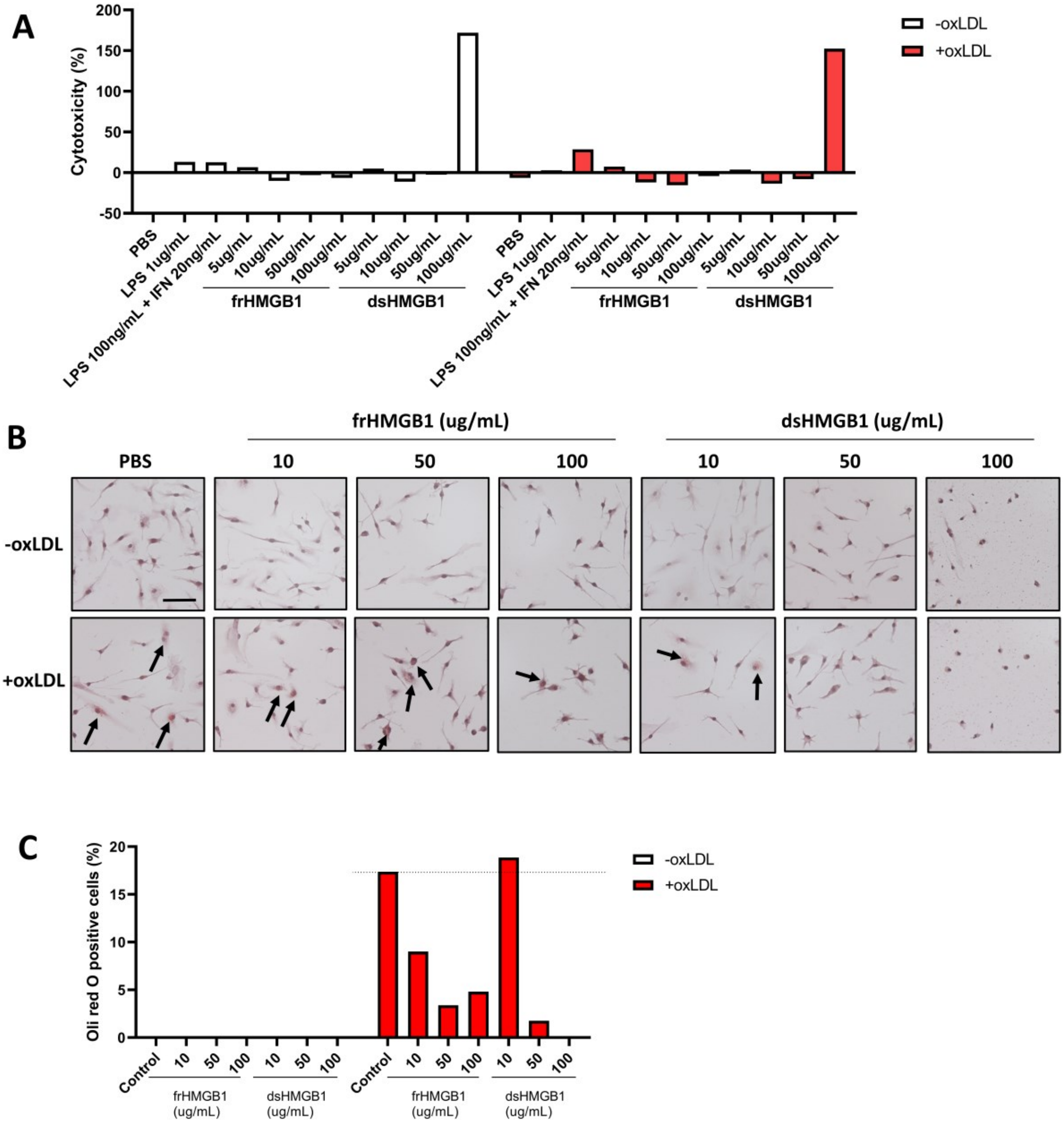

Supplementary Figure S3. Effects of HMGB1 on foam cell formation from BMDMs. After incubation with frHMGB1 and dsHMGB1 (10, 50 and 100 μg/mL), BMDMs were treated with oxLDL (100 μg/mL) for 24 h. (A) LDH cytotoxicity assay revealed that dsHMGB1 in 100ug/mL was cytotoxic to BMDMs. (B) Oil Red O staining in oxLDL-induced BMDM with pre-treatment. Scale bar= 10 μm. (C) The percentage of cells with lipid droplets stained using Oil Red O in BMDM with and without oxLDL induction, normalized with haematoxylin-stained nuclear. Three pictures were taken in each condition and average was calculated, n=1.

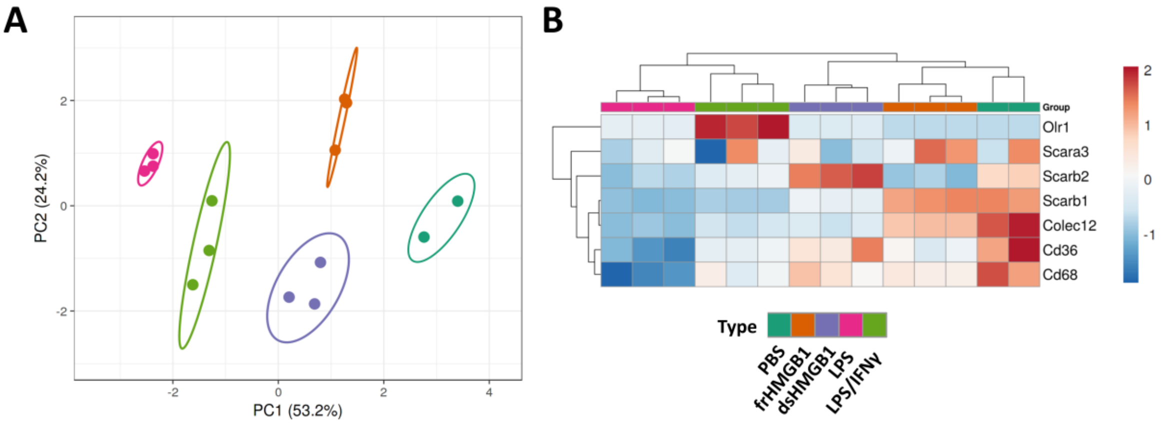

Supplementary Figure S4. Gene expression of scavenger receptors to oxLDL. (A) Principle Component plot based on the gene expression of seven scavenger receptors (*Scarb1*, *Scarb2*, *Cd36*, *Colec12*, *Scara3*, *Cd68* and *Olr1*) to oxLDL. (B) Heatmap was created based on the normalized gene counts in each sample. Colour intensity was scaled within each row so that the highest value corresponds to red and the lowest to blue.
